# Transcription Driven Somatic DNA Methylation within the Imprinted *Gnas* Cluster

**DOI:** 10.1371/journal.pone.0117378

**Published:** 2015-02-06

**Authors:** Stuti Mehta, Christine M. Williamson, Simon Ball, Charlotte Tibbit, Colin Beechey, Martin Fray, Jo Peters

**Affiliations:** 1 Mammalian Genetics Unit, MRC Harwell, Harwell Science and Innovation Campus, Oxfordshire, OX11 0RD, United Kingdom; 2 Mary Lyon Centre, MRC Harwell, Harwell Science and Innovation Campus, Oxfordshire, OX11 0RD, United Kingdom; University of Bonn, Institut of experimental hematology and transfusion medicine, GERMANY

## Abstract

Differential marking of genes in female and male gametes by DNA methylation is essential to genomic imprinting. In female gametes transcription traversing differentially methylated regions (DMRs) is a common requirement for *de novo* methylation at DMRs. At the imprinted *Gnas* cluster oocyte specific transcription of a protein-coding transcript, *Nesp*, is needed for methylation of two DMRs intragenic to *Nesp*, namely the *Nespas-Gnasxl* DMR and the *Exon1A* DMR, thereby enabling expression of the *Gnas* transcript and repression of the *Gnasxl* transcript. On the paternal allele, *Nesp* is repressed, the germline DMRs are unmethylated, *Gnas* is repressed and *Gnasxl* is expressed. Using mutant mouse models, we show that on the paternal allele, ectopic transcription of *Nesp* traversing the intragenic *Exon1A* DMR (which regulates *Gnas* expression) results in *de novo* methylation of the *Exon1A* DMR and de-repression of *Gnas* just as on the maternal allele. However, unlike the maternal allele, methylation on the mutant paternal allele occurs post-fertilisation, i.e. in somatic cells. This, to our knowledge is the first example of transcript/transcription driven DNA methylation of an intragenic CpG island, in somatic tissues, suggesting that transcription driven *de novo* methylation is not restricted to the germline in the mouse. Additionally, *Gnasxl* is repressed on a paternal chromosome on which *Nesp* is ectopically expressed. Thus, a paternally inherited *Gnas* cluster showing ectopic expression of *Nesp* is “maternalised” in terms of *Gnasxl* and *Gnas* expression. We show that these mice have a phenotype similar to mutants with two expressed doses of *Gnas* and none of *Gnasxl*.

## INTRODUCTION

**Fig 1:**
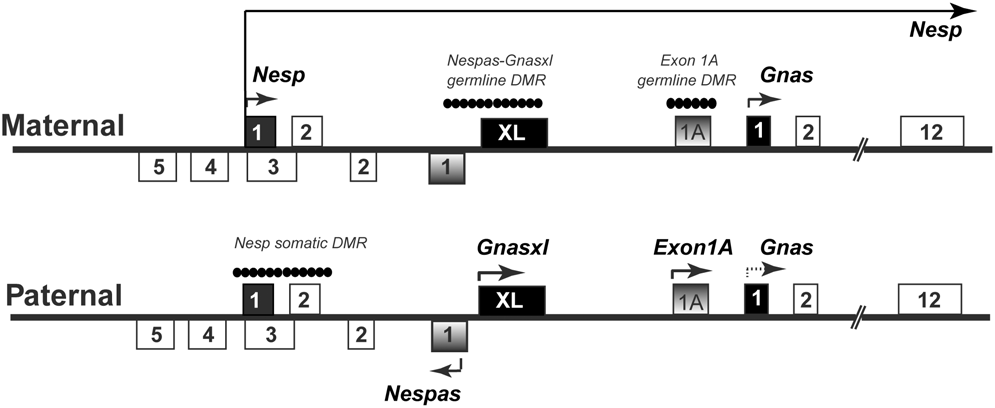
Organisation of the mouse *Gnas* locus. Both the maternally and paternally inherited copies of the *Gnas* cluster are shown. Boxes represent exons. The solid black filled boxes represent first exons of the protein-coding transcripts *Nesp*, *Gnas* and *Gnasxl* (labelled XL) whereas shaded boxes are first exons of the non-coding transcripts *Nespas* and *Exon1A* (labelled 1A). Arrows show the direction of transcription. *Gnas* expression is shown as a dotted line as *Gnas* itself shows tissue-specific imprinted expression. The position of the differentially methylated regions (DMRs) is shown by a string of filled circles on the allele on which the DMR is methylated. *Nesp* transcription traverses the entire length of the cluster, including the *Nespas-Gnasxl* DMR and the *Exon1A* DMR, as shown by a long arrow. The figure is not to scale. Adapted from Williamson *et al* (2011).

Genomic imprinting, which results in two genetically identical genes showing distinct expression patterns according to parental origin, has traditionally been a useful model system for studying epigenetic modification and processes. Nearly all imprinted genes discovered to date are organised in small clusters of 2–15 genes [1]. The *Gnas* cluster is well conserved between man and mouse, and contains a number of maternally, paternally and biallelically expressed transcripts. Four transcripts (*Nesp*, *Gnasxl*, *Exon1A*, and *Gnas*) arise from distinct promoters and contain a unique first exon each that splices onto a set of common downstream exons [2–4]. *Nesp* is maternally expressed and codes for the neuroendocrine secretory protein, NESP55 [3,5]. It originates furthest upstream and transcribes through the entire length of the cluster (Fig. 1). *Gnas* codes for the stimulatory G-protein Gsα, and also gives rise to a shortened neural form GsαN1. *Gnas* is biallelically expressed in most tissues bar a few, where it is preferentially maternally expressed [6]. *Gnasxl* is paternally expressed, codes for extra large forms of Gsα and gives rise to a number of different protein variants [7]. These comprise XLαs, an N-terminally extended form XXLαs and in neural tissues a C-terminally truncated form XLN1 [8]. In addition a protein called ALEX is generated from an alternative reading frame of the first exon, the XL exon of the *Gnasxl* transcript (Fig. 1). *Exon1A* is also paternally expressed and is a non-coding transcript [9]. A fifth transcript, *Nespas*, is a non-coding, paternally expressed transcript, which is anti-sense to *Nesp*. *Nespas* is transcribed in a direction opposite to all above transcripts, and covers the promoter region of *Nesp* alone (Fig. 1) [10,11].

Disturbance in imprinted expression of *Gnas* and *Gnasxl* largely accounts for the phenotypes observed in mice carrying mutations at the *Gnas* cluster [12]. Proteins Gsα and XLαs, which both form the alpha subunit of the heterotrimeric G protein perform antagonistic physiological functions [13]. Their imprinted expression, which gives rise to an approximate 1:1 balanced dosage of *Gnas* and *Gnasxl*, is required for normal postnatal growth and development in the mouse [12].

Imprinted expression of transcripts at the *Gnas* cluster is controlled by three regions of differential methylation (DMRs, Fig. 1). These comprise a somatic, paternally methylated DMR encompassing the promoter of *Nesp* [2,3]; and unusually, two germline DMRs both of which are intragenic to the *Nesp* transcription unit and are maternally methylated: the *Nespas*-*Gnasxl* DMR and the *Exon1A* DMR. The *Nespas*-*Gnasxl* DMR contains promoters of *Nespas* and *Gnasxl* both of which are silent on the maternal allele [14]. The *Exon1A* DMR controls the downstream *Gnas* promoter and the imprinted expression of *Gnas* [4,15].

Methylation of the germline DMRs on the maternal allele is acquired in the oocyte, where acquisition of *de novo* methylation requires expression of *Nesp* to traverse both the downstream DMRs [16]. In contrast, on the paternal allele, such germline methylation is absent, resulting in an opposite pattern of expression for *Gnasxl* and *Gnas* on the paternal allele. Post fertilisation, paternal *Nespas* expression silences *Nesp* on the paternal allele and the *Nesp* DMR gains methylation [17].

We hypothesised that the driving force behind *Nespas* expression induced silencing of *Nesp* on the paternal allele is the competency of *Nesp* expression to induce silencing of *Gnasxl* but enable expression of *Gnas* on the paternal allele. From previous work, we know that ectopic expression of *Nesp* on the paternal allele results in a drastic reduction in *Gnasxl* expression but in the absence of *de novo* methylation at the *Nespas*-*Gnasxl* DMR [17]. However the effects of paternal expression of *Nesp* on *Gnas* expression were not known. In this study we have used two mutants in which *Nesp* is de-repressed on the paternal allele. We investigated if expression of *Nesp* traversing the *Exon1A* DMR results in gain of *de novo* methylation at the paternal *Exon1A* DMR, and de-repression of *Gnas* on the paternal allele. Our results show that on the paternal allele, *Nesp* expression traversing through the *Exon1A* DMR results in acquisition of *de novo* methylation at the *Exon1A* DMR, just as it does on the maternal allele. However, *Nesp* expression induced *de novo* methylation of the *Exon1A* DMR occurs post-fertilization, in contrast to the wildtype maternal *Nesp* expression driven *de novo* methylation that occurs in the oocyte [16]. DMRs are CpG rich, and hence a subset of CpG islands (CGIs). Presence of methylated intragenic CGIs is an established feature of actively transcribed genes in somatic cells of various eukaryotes [18,19]. In recent years, upstream originating transcription has emerged as a major driver of methylation of intragenic CGIs in the oocyte, including at many germline DMRs [16,20–22]; however, it was not known if a causal relationship exists between transcription and intragenic/gene-body methylation in somatic cells. To our knowledge, ectopic *Nesp* expression driven gain of methylation at the intragenic *Exon1A* DMR is the first example of transcription driven *de novo* methylation in somatic cells.

As a result of ectopic *de novo* methylation of the *Exon1A* DMR, *Gnas* expression is upregulated in mutant mice. Thus paternal *Nesp* transcription leads to ‘maternalisation’ of the paternal allele, giving rise to an imbalance in the total expressed doses of *Gnas* and *Gnasxl*. Furthermore, mutant mice show a phenotype remarkably similar to mice with maternal duplication of distal 2 (MatDp(dist2)), which have two expressed doses of maternally inherited *Gnas*, and no expressed dose of *Gnasxl* [23].

## MATERIAL AND METHODS

### Mouse breeding

All mouse studies were conducted under guidance issued by the Medical Research Council in ‘Responsibility in the Use of Animals in Bioscience Research’ (May 2008) and under the authority of Home Office Project Licence Numbers 30/2065 and 30/2526. For the characterisation of +/*T* ^*ex1*^ and +/*T* ^*int2*^, mice were examined daily and observations recorded using a numerical system on a welfare scoring sheet. From birth onwards animals were scored for up to 12 parameters affecting feeding, growth, morphology and activity. Humane endpoints such as pale appearance leading to cyanosis and failure to feed and/or thrive were used and animals reaching a humane endpoint were humanely sacrificed either by a schedule one method authorised by UK A(SP)A legislation or a non-schedule one method authorised under project licences 30/2065 and 30/2526. Increased monitoring regimes by trained and competent animal care staff were put in place in order to identify welfare problems and intervene at the earliest relevant timepoint.

Mice were housed in Tecniplast IVC 1284L caging with a maximum number of 5 mice per cage. All cages contained pine bedding (Datesand grade 6) and Datesand rodent tunnels and shredded paper for environmental enrichment. All mice had free access to water and diet [Special diet services(Dietex) RM3 (E)] in a 12-hour light-dark cycle with room temperature 19–22°C.

**Fig 2:**
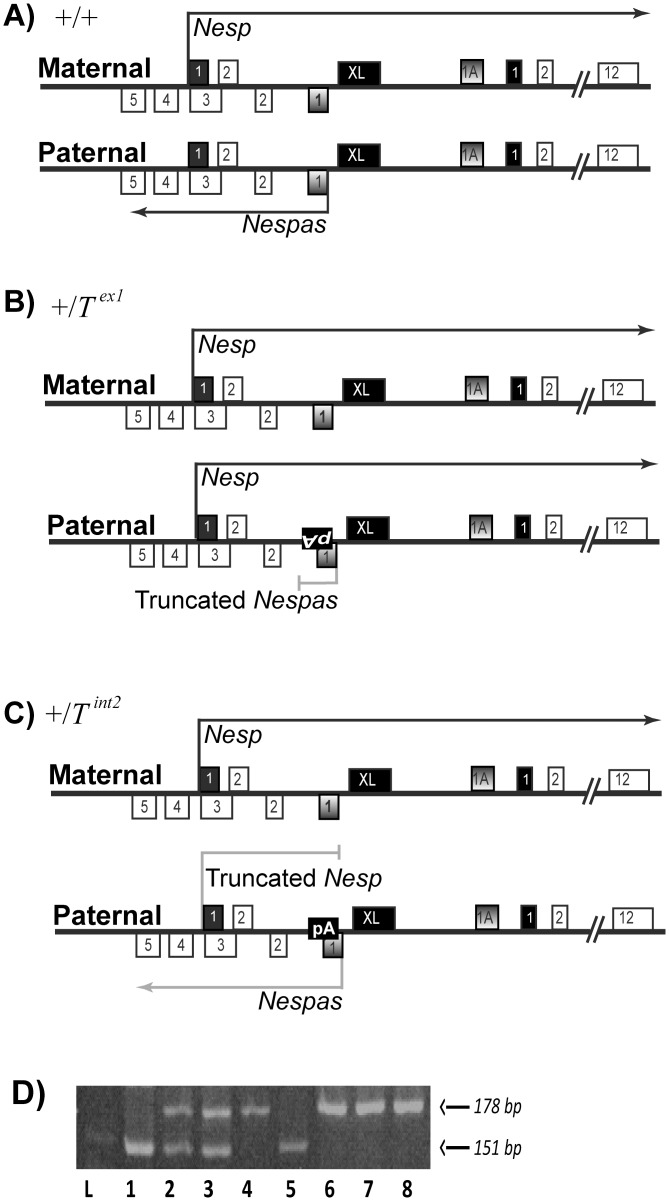
Schematic of the *Gnas* cluster in (A) +/+ (B) *+/T* ^ex1^ and (C) *+/T* ^int2^ mice. Solid black fill boxes represent first exons of the protein-coding transcripts *Nesp*, *Gnas* and *Gnasxl* (XL) whereas shaded boxes are first exons of the non-coding transcripts *Nespas* and *Exon1A* (1A). (B) Insertion of a poly-A cassette is shown as inverted ‘pA’ on the paternal *T* ^*ex1*^ allele. On this allele, *Nespas* was truncated, and *Nesp* was de-repressed. (C) The poly-A cassette is inserted in the reverse orientation (shown as pA) on the paternal *T* ^*int2*^ allele. On this allele, *Nespas* was not truncated but was expressed at a low level (low expression shown as grey arrow), and *Nesp* was de-repressed and truncated. (D) *Nesp* is expressed biallelically at 10.5 dpc in *SD2/T* ^*ex1*^ embryos. *Nesp* expressed from an SD2 allele shows a band of 151bp, and *Nesp* from a *T* ^*ex1*^ (129/*SvEv* inbred strain) allele band shows a band of 178bp upon digestion with *Bst*UI. Lanes 2,3 of *SD2/T* ^*ex1*^ mutants show bands of both sizes, indicating that *Nesp* is expressed from both parental alleles at 10.5 dpc. Lane 1 shows 10.5 dpc *SD2*/+; Lane 4, 129*SvEv* wild-type neonatal brain; Lane 5, SD2 neonatal brain; Lane, 6,7, 10.5 dpc *T* ^*ex1*^/*SD2*; Lane 8, 10.5 dpc +/*SD2*; Lane 1 is a DNA ladder.

The generation of the mutant alleles *T* ^*ex1*^ and *T* ^*int2*^ has been described previously [17], where *T* ^*ex1*^ was designated as *Nespas-T* ^*ex1*^ (MGI ID:4950066) and *T* ^*int2*^ as *Nesp-T* ^*int2*^ (MGI ID:4950063). Briefly, a polyadenylation cassette from the rabbit β-globin gene was inserted into exon 1 of *Nespas* between nucleotides 151519 and 151520 of AL593857.10 in an orientation that truncated *Nespas* in *T* ^*ex1*^ (labelled as an inverted pA in Fig. 2B) and truncated *Nesp* in *T* ^*int2*^ (labelled pA in Fig. 2c). Both mutations were maintained as heterozygotes on an inbred 129/*SvEv* strain. The *ΔExon1A* mutation is a deletion of the *Exon1A* DMR [4] and was maintained as a homozygous stock on 129/*SvEv*. Compound heterozygous mice (all genotypes are represented as maternal allele/paternal allele): +/*T* ^*ex1*^; *ΔExon1A*/+, +/+; *ΔExon1A*/+, +/*T* ^*int2*^; *ΔExon1A*/+, +/+; *ΔExon1A*/+ were generated for methylation analysis by bisulfite modification and by restriction sensitive Southern blotting. To produce +/*T* ^*ex1*^; *ΔExon1A*/+ and +/+; *ΔExon1A*/+ mice, heterozygous *T* ^*ex1*^/+ males were crossed with homozygous *ΔExon1A/ ΔExon1A* females. The offspring were of genotype +/*T* ^*ex1*^; *ΔExon1A*/+ and +/+; *ΔExon1A*/+. Similarly, to generate +/*T* ^*int2*^; *ΔExon1A*/+ and +/+; *ΔExon1A*/+ mice, *T* ^*int2*^/+ males were crossed with *ΔExon1A*/ *ΔExon1A* females. A reciprocal cross, with *ΔExon1A*/ *ΔExon1A* males and *T* ^*int2*^/+ females was set up to generate *T* ^*int2*^/+; +/ *ΔExon1A* and +/+; +/ *ΔExon1A* offspring.

To analyse *Exon1A* DMR methylation on the chromosome carrying the *T* ^*ex1*^ allele in sperm, heterozygous *T* ^*ex1*^/+ females were crossed with homozygous *ΔExon1A/ ΔExon1A* males to generate compound heterozygotes *T* ^*ex1*^/+; +/ *ΔExon1A*. Males of this genotype are expected to produce sperm of *T* ^*ex1*^ *Exon1A* or + *ΔExon1A* genotypes. Mice were genotyped for the *T* ^*ex1*^ and *T* ^*int2*^ alleles by PCR as described previously [17].

Whole embryos at 10.5 dpc (+/*T* ^*ex1*^; *ΔExon1A*/+ and +/+; *ΔExon1A*/+) were collected by counting the day of sighting a vaginal plug as 0.5 dpc. Sperm was collected from *T* ^*ex1*^/+; +/ *ΔExon1A* and +/+; +/ *ΔExon1A* adult littermate males at six to nine weeks of age. Mice were sacrificed and sperm squeezed out from the vas deferens and epidydimis into 500 μl freshly prepared lysis 1 solution [75 mM NaCl, 25 mM EDTA (pH 8.0) and 10 μl/ml β-mercaptoethanol (Sigma)] and washed twice in cold PBS (137 mM NaCl, 10 mM Phosphate, 2.7 mM KCl, pH 7.4) before DNA extraction.

### Methylation analyses

Sperm collected in 500 μl lysis 1 were mixed with 500 μl of freshly prepared lysis solution 2 [10 mM Tris-HCl (pH 8.0), 10 mM EDTA (pH 8.0), 1% SDS] and incubated at 55°C for one hour to lyse sperm heads. Proteinase K was then added to a final concentration of 400 μg/ml and incubated overnight at 55°C. DNA was extracted by performing standard phenol-chloroform-isoamyl alcohol (25:24:1, v:v:v) and chloroform-isoamyl alcohol (24:1, v:v) extractions. Genomic DNA was extracted from neonatal brain and from whole 10.5 dpc embryos with an Allprep DNA/RNA Mini Kit (Qiagen). Bisulfite conversion was performed on genomic DNA (1 μg from neonatal brain and whole embryos, entire amount from sperm) with an Epitect kit (Qiagen), and a 327bp region of the *Exon1A* DMR corresponding to nucleotides 183866–184147 of AL593857.10 was PCR amplified as described previously [24]. Sequences were analysed using the BiQ analyser programme [25] and only unique clones that showed >80% similarity between the experimental and the genomic sequence and >90% non-CpG C→T conversion were included in the analysis.

Methylation sensitive Southern blot analysis on newborn brain was performed as described before [24]. Briefly, genomic DNA (from +/*T* ^*ex1*^; *ΔExon1A*/+, +/+; *ΔExon1A*/+, +/*T* ^*int2*^; *ΔExon1A*/+) was digested with *Bam*HI (-), *Bam*HI and *Hpa*II (H), and *Bam*HI and *Msp*I (M). A 1.9 kb *Bam*HI—*Bg*lII fragment that encompasses the *Exon1A* DMR was used as a probe.

### RNA analysis

Frozen tissue was homogenised using a rotor-stator Ultraturra Basic T25 Homogeniser (Labortechnik). Total RNA was extracted from newborn brain with the Allprep DNA/RNA Mini Kit (Qiagen) and from Brown Adipose Tissue (BAT) with an RNAeasy lipid kit (Qiagen), followed by reverse transcription with the High Capacity cDNA Reverse Transcription kit (Applied Biosystems). RNA was treated with RQ1 RNase-free DNase (Qiagen) to remove traces of contaminating genomic DNA.

The relative quantification of *Gnas*, *Gnasxl* and *Exon1A* transcripts was carried out by quantitative real time PCR (qPCR). Each reaction contained 1x pre-assembled Taqman gene expression assay (a transcript specific FAM dye labelled TaqMan MGB probe and an unlabelled primer set), 1x TaqMan Fast Universal PCR Master Mix (Applied Biosystems) and 50 ng of cDNA, and was performed in triplicate on a 7500 Fast Real-Time PCR machine. The amounts of *Gnas*, *Gnasxl* and *Exon1A* transcripts were normalised to the reference gene glyceraldehyde 3-phosphate dehydrogenase (*Gapdh*), and difference in expression between mutant and control samples was determined using the comparative C_T_ (threshold cycle) method as described previously [17]. Northern blots were performed as described previously [24] using actin beta (*Actb*) as a loading control.

Detection of the allelic origin of *Nesp* at 10.5 dpc was performed as described earlier [24]. Reciprocal crosses between *T* ^*ex1*^/+ and SD2 mice were performed. SD2 are of a predominantly *Mus musculus* genetic background, but carry the distal portion of chromosome 2 derived from *Mus spretus*. Consequently, the SD2 have a *BstU*1 site in exon12 of *Gnas* that the 129*SvEv* (*Mus musculus*) do not. *Nesp* was amplified by RT-PCR from 10.5 dpc embryos of genotype SD2/*T* ^*ex1*^, SD2/+, the reciprocal *T* ^*ex1*^/SD2, +/SD2 as well as from neonatal brain of SD2 and 129*SvEv* that acted as controls. RT-PCR products were digested by *BstU*1, which gives products of 151bp for *Nesp* derived from a *M*. *spretus* allele and 178bp products derived from a *M*. *musculus* allele.

### Mouse weights

Mice were weighed daily from 17.5 dpc until postnatal day 29 (day of birth called P0). The average weight of wild-types was calculated at each time point. Then the weight of each individual mouse was taken as a percentage of the average wild-type weight at each time point.

### Statistical Analysis

Fisher’s exact test was used for comparison of the incidence of +/*T* ^*ex1*^ and +/*T* ^*int2*^ and for assessing the results of the suckling observations. Student’s *t* test (two-tailed) was used for evaluating the weight studies and the quantitation of transcripts at the *Gnas* cluster.

## RESULTS

### The *Exon1A* DMR is completely methylated when *Nesp* is transcribed through the *Gnas* cluster on the paternal allele

**Fig 3:**
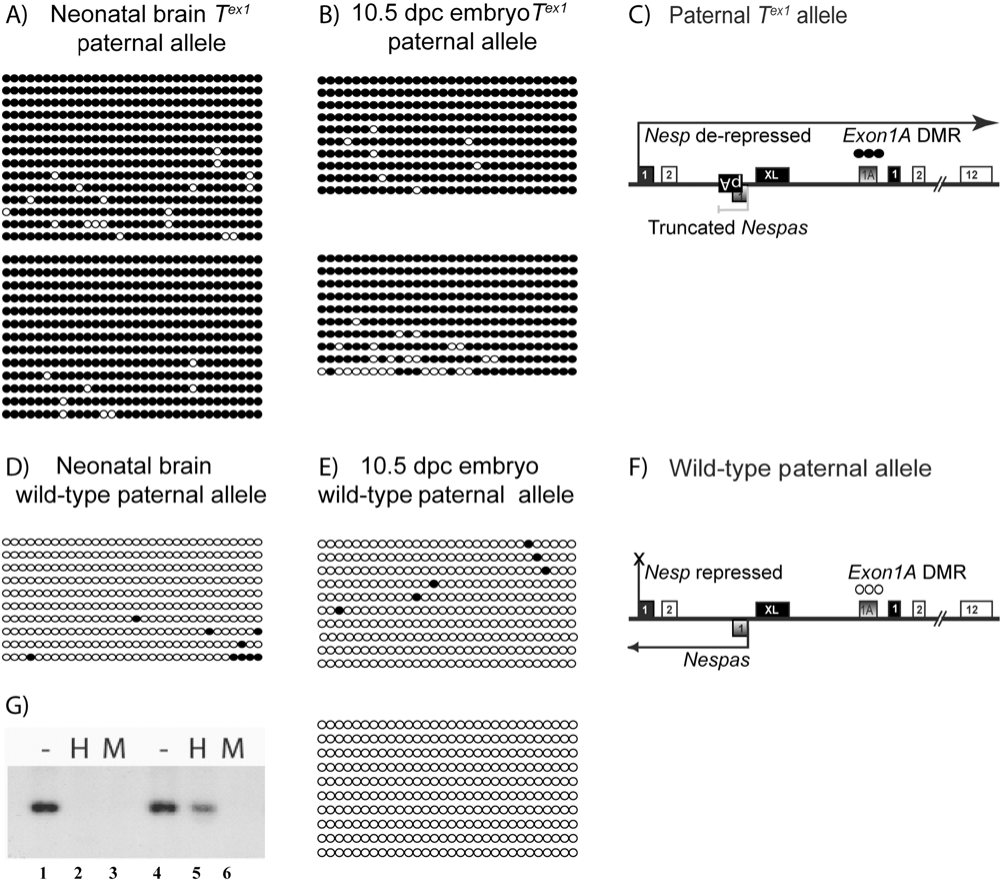
Methylation of the *Exon1A* DMR on paternal inheritance of the *T* ^ex1^ allele in (A) neonatal brain, n = 2 and (B) 10.5 dpc embryos, n = 2, both of the +/*T* ^ex1^; *ΔExon1A*/+ genotype. Each circle represents a CpG dinucleotide; filled when methylated and open when unmethylated. Each string of circles is a unique clone, and all clones from an individual are grouped into a block. (C) shows a summary of *Nespas* and *Nesp* expression and *Exon1A* DMR methylation on paternal inheritance of a *T* ^*ex1*^ allele. The solid black filled boxes represent first exons of the protein-coding transcripts *Nesp*, *Gnas* and *Gnasxl* whereas shaded boxes are first exons of the non-coding transcripts *Nespas* and *Exon1A*. Hypomorphic *Nespas* expression is shown in grey. (D) Methylation of the paternally inherited *Exon1A* DMR in control littermates +/+; *ΔExon1A*/+ in neonatal brain, n = 1 and in (E) 10.5 dpc embryos, n = 2. (F) shows a summary of *Nespas* and *Nesp* expression and *Exon1A* DMR methylation on a wild-type paternal allele. (G) shows a methylation sensitive Southern blot performed on +/*T* ^*ex1*^; *ΔExon1A*/+ (lanes 4,5,6) and +/+; *ΔExon1A*/+ (lanes 1,2,3) neonatal brains. *Bam*HI digestion (-), *Bam*HI and *Hpa*II (H), and *Bam*HI and *Msp*I (M) digestions probed for the *Exon1A* DMR are shown for each sample. Sample +/*T* ^*ex1*^; *ΔExon1A*/+ resists complete digestion by the restriction sensitive *Hpa*II (lane 5) suggesting methylation at the *Exon1A* DMR.

We first examined the methylation status of the *Exon1A* DMR in neonatal brain and in 10.5 dpc whole embryos carrying a paternally inherited *Nespas-T* ^*ex1*^ allele (henceforth, *T* ^*ex1*^). *T* ^*ex1*^ is a truncation allele of *Nespas*, generated by the insertion of a poly-A cassette in *Nespas* exon1 [17] (Fig. 2B). On paternal inheritance of the *T* ^*ex1*^ allele, *Nesp* was fully de-repressed and transcribed through the entire length of the cluster, including the *Exon1A* DMR in neonatal brain [17] and at 10.5 dpc (Fig. 2B, 2D). Using bisulfite sequencing, we investigated the methylation status of a representative 327bp CpG-rich region of the *Exon1A* DMR on paternal inheritance of this allele. To ensure that only the paternally inherited *Exon1A* DMR was amplified in the PCR following bisulfite modification, compound heterozygotes +/*T* ^*ex1*^; *ΔExon1A*/+ were used. In addition to a paternally derived *T* ^*ex1*^ allele, these mice carry a maternally derived deletion allele of the *Exon1A* DMR, named *ΔExon1A* [4]. Thus, only the paternally inherited *Exon1A* DMR was available for amplification and analysis. Earlier work has shown that a monoallelic deletion of the *Exon1A* DMR *per se* does not result in a change in the methylation status of the other, intact *Exon1A* DMR implying that in our mutants, deletion of the maternal *Exon1A* DMR does not have a *trans* effect on the imprinting status of the paternal *Exon1A* DMR [4,15]. The analysed region of the *Exon1A* DMR was almost completely methylated on the *T* ^*ex1*^ *Exon1A* ^+^ allele in neonatal brain and in 10.5 dpc embryos (Fig. 3 A, B, C). As expected, the *Exon1A* DMR was almost completely unmethylated on the paternal allele in littermate controls (Fig. 3 D, E, F). The results from neonatal brain were confirmed by methylation sensitive Southern blotting analysis (Fig. 3G).

Thus, on paternal inheritance of the *T* ^*ex1*^ allele, *Nesp* is transcribed through the *Exon1A* DMR and the latter is completely methylated.

### The gain of ectopic paternal methylation at the *Exon1A* DMR is a somatic event

**Fig 4:**
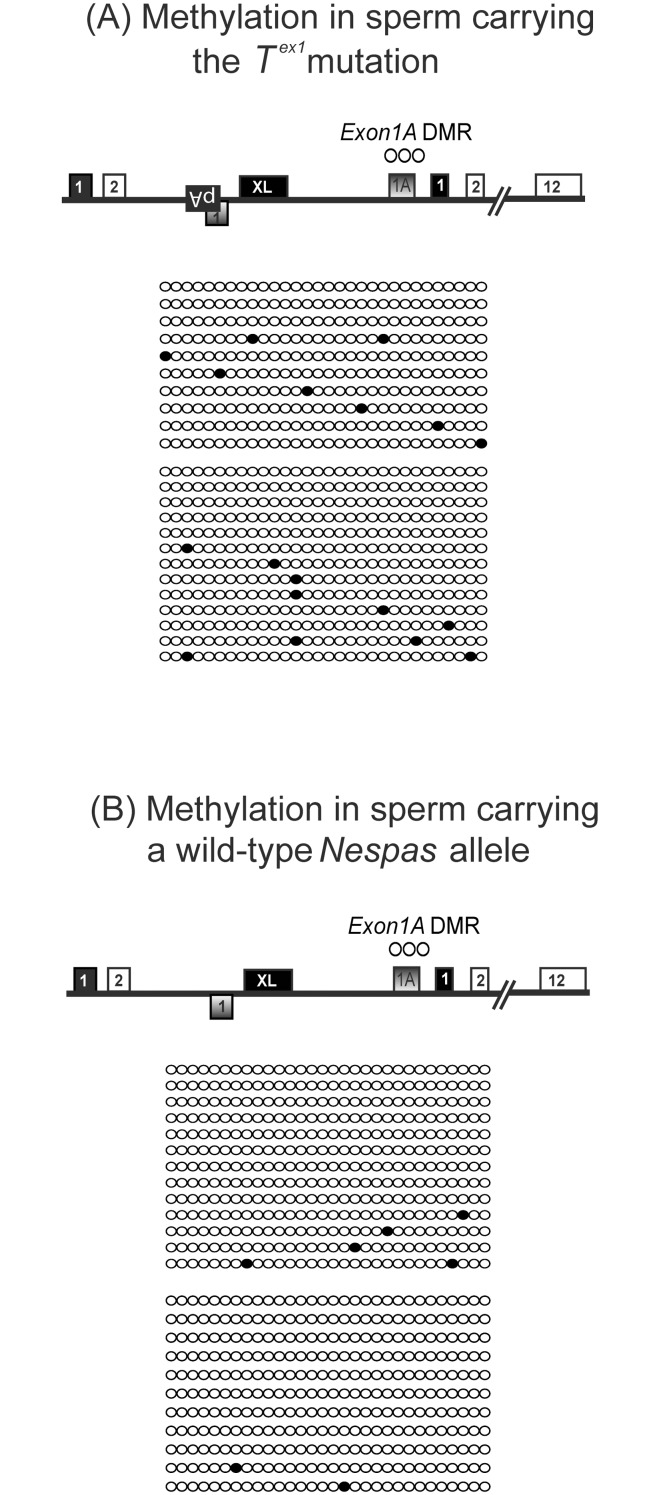
Methylation of the *Exon1A* DMR in sperm. (A) *Exon1A* DMR methylation in sperm of mice carrying the *T* ^*ex1*^ mutation (of genotype *T* ^*ex1*^/+; +/Δ*Exon1A*, n = 2). (B) *Exon1A* DMR methylation in sperm of littermate control males (of genotype +/+; +/Δ*Exon1A*, n = 2).

We next examined if the methylation at the *Exon1A* DMR on paternal inheritance of the *T* ^*ex1*^ allele was acquired in the germline; reminiscent of the gain of *de novo* methylation at the *Exon1A* DMR in the oocyte. *De novo* methylation of paternally methylated germline DMRs normally begins around 14.5 dpc, is complete by the neonatal stage and can be detected in mature sperm [26–28]. Bisulfite analysis of sperm from *T* ^*ex1*^ carriers (*T* ^*ex1*^/+; +/ *ΔExon1A*) revealed a virtually unmethylated *Exon1A* DMR, akin to the *Exon1A* DMR in sperm of littermate control mice (Fig. 4). We conclude that ectopic methylation of the *Exon1A* DMR on paternal inheritance of the *T* ^*ex1*^ allele is acquired post-fertilisation and therefore is a somatic mark, and not a germline mark.

### When *Nesp* is expressed on the paternal allele but truncated upstream of the *Exon1A* DMR, the *Exon1A* DMR is not methylated

**Fig 5:**
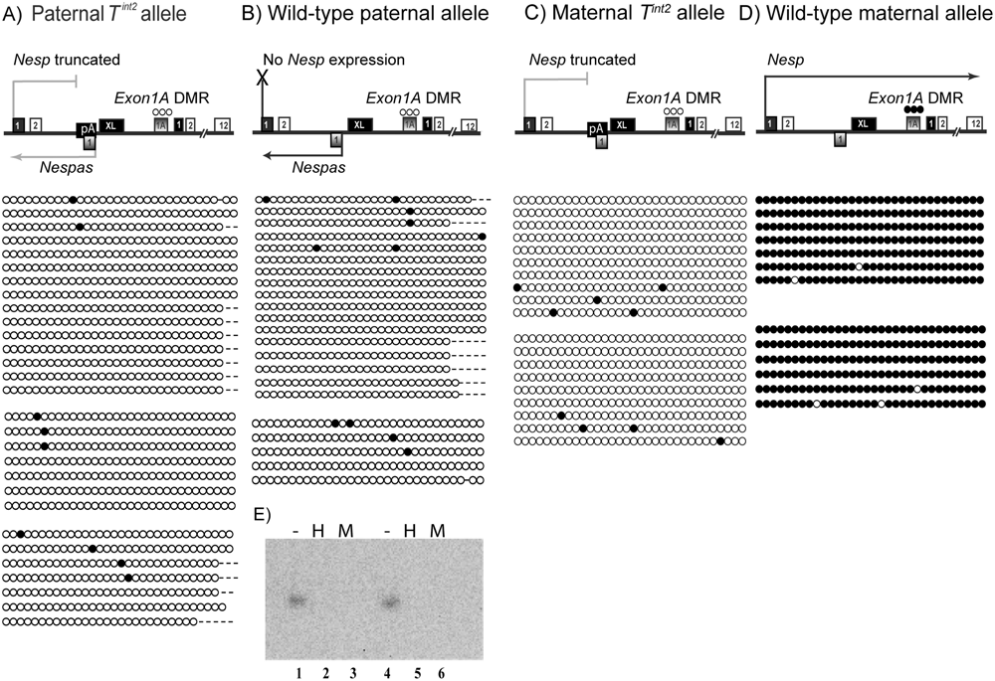
Methylation of the *Exon1A* DMR on the *T* ^int2^ *Exon1A* ^+^ allele in neonatal brain. Upper panels show a schematic of the wildtype *Gnas* cluster or that of the *Gnas* cluster carrying a *T* ^*int2*^ mutant allele. Lower panels show corresponding bilsulfite methylation profiles of the *Exon 1A* DMR. (A) Upper panel: Line drawing of the *Gnas* cluster on paternal inheritance of the *T* ^*int2*^ allele. Lower panel: Bisulfite methylation profile of the paternal *Exon1A* DMR in neonatal brain of genotype *+/T* ^*int2*^ *; ΔExon1A*/ +, n = 3. A ‘-’ shows no result at that CpG. (B) Upper panel: Line drawing of a paternally inherited wild-type *Gnas* cluster. Lower panel: Bisulfite methylation profile of the paternal *Exon1A* DMR in neonatal brain of littermate controls, *+/+; ΔExon1A*/ +, n = 2. (C) Upper panel: Line drawing of the *Gnas* cluster on maternal inheritance of the *T* ^*int2*^ allele. Lower panel: Bisulfite methylation profile of the maternal *Exon1A* DMR in neonatal brain of *T* ^*int2*^/+;+/*ΔExon1A*, n = 2. (D) Upper panel: Line drawing of a maternally inherited wild-type *Gnas* cluster. Lower panel: Bisulfite methylation profile of the maternal *Exon1A* DMR in neonatal brain of littermate controls, *+/+;+/ΔExon1A*, n = 2. (E) Methylation sensitive Southern blot performed on +/*T* ^*int2*^; Δ*Exon1A*/+ (lanes 4,5,6) and +/+; Δ*Exon1A*/+ (lanes 1,2,3) neonatal brains. *Bam*HI digestion (-), *Bam*HI and *Hpa*II (H), and *Bam*HI and *Msp*I (M) digestions probed for the *Exon1A* DMR are shown for each sample. Both samples are completely digested by the restriction sensitive *Hpa*II suggesting absence of methylation at *Hpa*II sites in the *Exon1A* DMR.

Next we examined the methylation status of the *Exon1A* DMR when a *Nesp-T* ^*int2*^ allele (*T* ^*int2*^ henceforth) is paternally inherited. *T* ^*int2*^ was generated by insertion of a poly-A cassette at the same position as in *T* ^*ex1*^, but in a reverse orientation (Fig. 2C). As a result, the insertion no longer truncated *Nespas*, and was expected to truncate *Nesp*. As previously described [17], paternal inheritance of *T* ^*int2*^ gave rise to a *Nespas* hypomorph, and to a low level expression of *Nesp* from the paternal allele. In addition, paternally expressed *Nesp* is truncated upstream to the *Exon1A* DMR in this mutant [17]. Bisulfite analysis of neonatal brain from compound heterozygotes +/*T* ^*int2*^; *ΔExon1A*/+ showed that the *Exon1A* DMR region on the *T* ^*int2*^ *Exon1A* ^+^ allele was not methylated (Fig. 5A). As expected, the *Exon1A* DMR was also unmethylated on the paternal allele in littermate control mice (Fig. 5B). These results were confirmed by methylation sensitive Southern blotting analysis (Fig. 5E).

Thus, on the paternal chromosome carrying the *T* ^*int2*^ allele, *Nesp* is expressed at low levels but its transcription does not traverse the *Exon1A* DMR, and the latter remains unmethylated.

### When the *T* ^*int2*^ mutation is transmitted maternally, the *Exon1A* DMR is unmethylated

When the *T* ^*int2*^ mutation is maternally inherited, the *Nesp* transcript expressed on the maternal allele is truncated, and *Nesp* expression is significantly reduced in brain of *T* ^*int2*^/+ neonates compared to that of wild-type siblings [17]. The *Exon1A* DMR region was predominantly unmethylated on the maternally inherited *T* ^*int2*^ *Exon1A* ^+^ allele in *T* ^*int2*^/+; +/*ΔExon1A* neonatal brain, whereas per expectation, the *Exon1A* DMR was extensively methylated on the maternal allele in littermate controls (Fig. 5 C, D).

### Imprinted expression of *Exon1A*, *Gnas* and *Gnasxl* is disrupted in +/*T* ^*ex1*^ and +/*T* ^*int2*^ mutants

**Fig 6:**
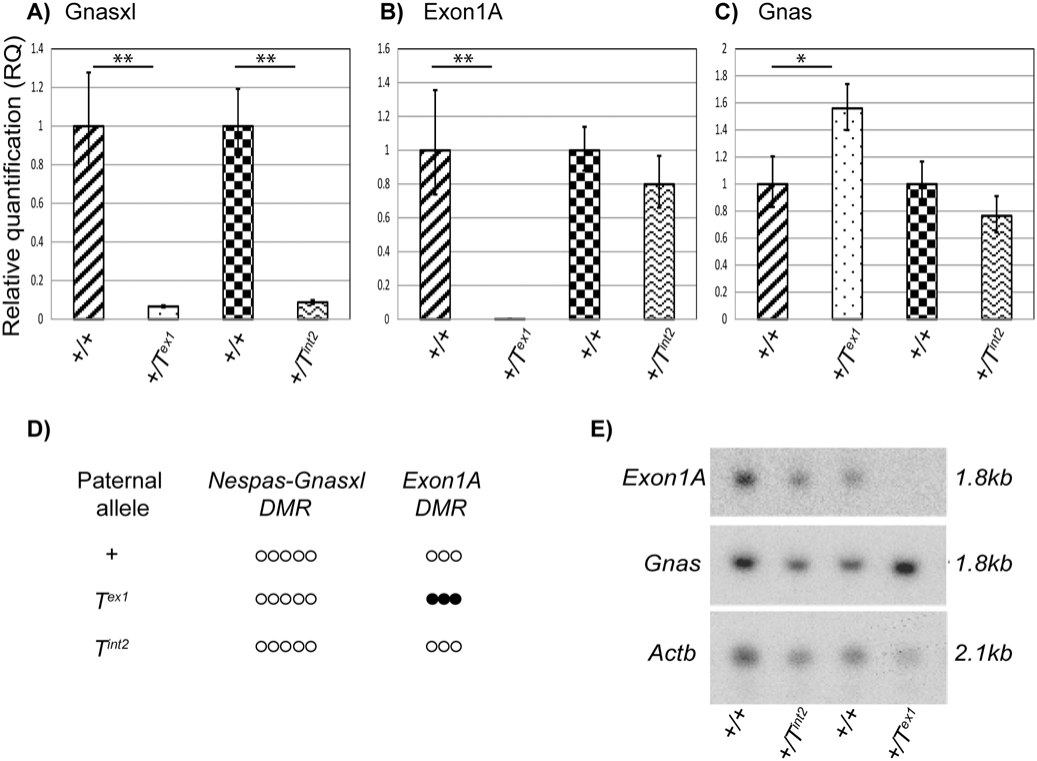
Expression of *Gnas*, *Gnasxl* and *Exon1A*, and methylation status of the *Nespas-Gnasxl* and *Exon1A* DMRs. Bar-charts (A, B, C) show relative quantification (RQ) of *Gnasxl*, *Gnas* and *Exon1A* transcripts in +/*T* ^*int2*^ and +/*T* ^*ex1*^ neonatal tissue compared to +/+ siblings (left). Error bars represent the range of possible RQ values defined by the standard error of the ΔCTs. * *p< 0*.*05*, ** *p<0*.*01*, as determined by a w-sample *t*-test. (D) Summary of methylation at the *Nespas-Gnasxl* DMR and the *Exon1A* DMR on paternal inheritance of a wild-type, *T* ^*ex1*^ and *T* ^*int2*^ allele. (E) Northern blot analysis of expression of *Exon1A* and *Gnas* in brown adipose tissue of +/*T* ^*int2*^ and +/*T* ^*ex1*^ neonates and their wild-type siblings. *Actb* is an endogenous loading control.

We wanted to determine the effect that ectopic methylation of the *Exon1A* DMR has on expression of *Exon1A* and *Gnas*. To investigate this, we measured *Exon1A* and *Gnas* transcripts in +/*T* ^*ex1*^ compared to wild-type littermates. Neonatal brown adipose tissue (BAT) in which *Gnas* shows imprinted expression was analysed [4]. Negligible amounts of *Exon1A* transcripts were detected in +/*T* ^*ex1*^ neonates, compared to those in wild-type littermates, using Taqman RT-PCR (~ 0.012% of wild-type; n = 5; *p =* 0.5 x 10^–6^, Fig. 6B). We also detected significantly higher amounts of *Gnas* transcripts in +/*T* ^*ex1*^ neonates compared to wild-type littermates (n = 4; *p =* 0.0158, Fig. 6C). The additional amount of *Gnas* must be expressed from the mutant paternal chromosome. No significant change was detected in the amount of *Exon1A* (n = 6; *p =* 0.099) or *Gnas* (n = 4; *p =* 0.094) in BAT of +/*T* ^*int2*^ neonates compared to wild-type littermates (Fig. 6 B, C). These results were supported by Northern blot analysis of total RNA from +/*T* ^*ex1*^and +/T^*int2*^ mice (Fig. 6E).

These results are consistent with the methylation status of the paternal *Exon1A* DMR in +/*T* ^*ex1*^and +/*T* ^*int2*^ neonates (Fig. 6D) and support the hypothesis that on a paternal chromosome expression of *Nesp* traversing the *Exon1A* DMR results in *de novo* methylation of the *Exon1A* DMR, and in turn leads to de-repression of *Gnas*.

We previously showed that the *Nespas-Gnasxl* DMR remained unmethylated on paternal inheritance of the *T* ^*ex1*^ or *T* ^*int2*^ mutant alleles [17]. Despite the lack of methylation, the levels of *Gnasxl* expression from both mutant alleles were drastically reduced [17]. Using a Taqman RT-PCR assay, we confirmed that the amount of *Gnasxl* was significantly lower in +/*T* ^*ex1*^ neonatal brain (6.6% of wild-type; n = 4; *p =* 0.021 x 10^–2^) when compared to wild-type littermates, and was also reduced in +/*T* ^*int2*^ mice (8.8% of wild-type; n = 4; *p =* 0.014 x 10^–5^, Fig. 6A).

Thus paternal inheritance of *T* ^*ex1*^ results in upregulation of *Gnas* and downregulation of *Gnasxl* whereas paternal transmission of *T* ^*int2*^ leads only to down regulation of *Gnasxl*.

### Anomalous phenotypes in +/*T* ^*ex1*^ and +/*T* ^*int2*^

Loss of *Gnasxl* is known to result in poor suckling, neonatal lethality and restricted growth; and upregulation of *Gnas* is associated with postnatal growth retardation [12]. We therefore investigated the phenotypes of both +/*T* ^*ex1*^ and +/*T* ^*int2*^.

**Fig 7:**
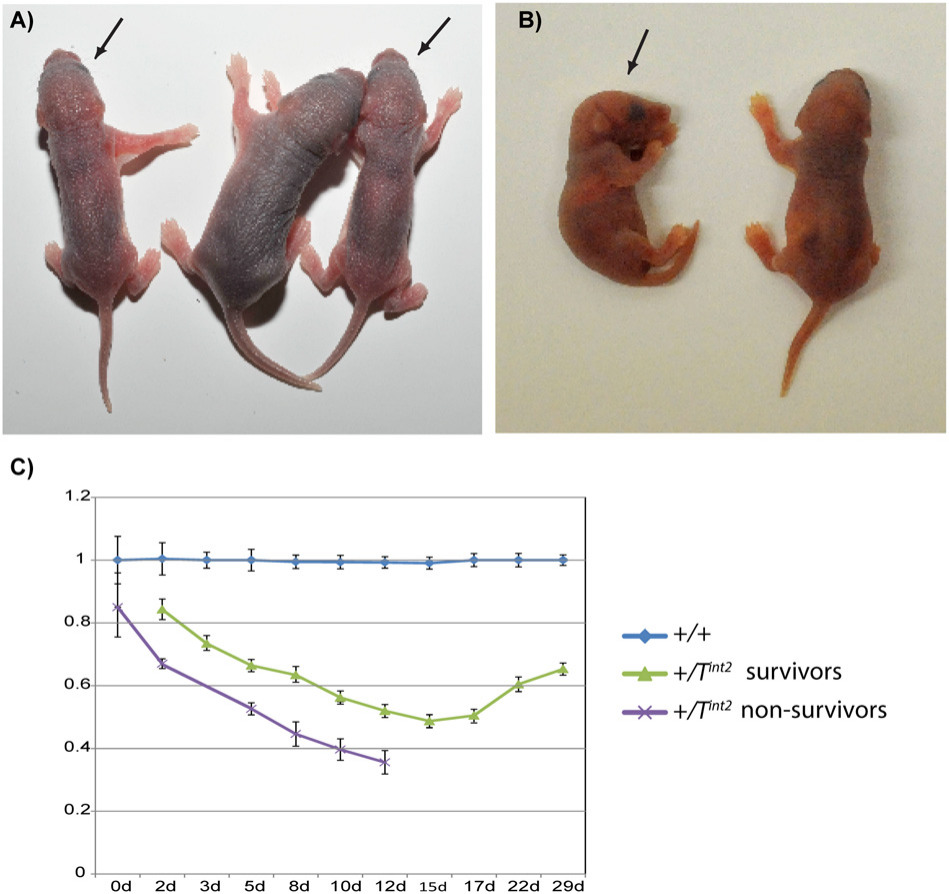
Phenotype of +/*T* ^int2^ and +/*T* ^ex1^. Appearance of *+/T* ^*int2*^ and +/*T* ^*ex1*^ (A) Two +/*T* ^*int2*^ and a wild-type sibling at postnatal day 2. The +/*T* ^*int2*^ (labelled with an arrow) are smaller and leaner than wild-type. (B) A newborn +/*T* ^*ex1*^ (labelled with an arrow) and a wild-type sibling. (C) Growth retardation. Shown is the growth curve of surviving and non-surviving +/*T* ^*int2*^ and wild-type littermates from 1 to 29 days post birth. The mean weight of wild-type littermates at each time-point have been normalised to 1 and the weights of +/*T* ^*int2*^ mice have been taken as a percentage of wild-type weights (n = 19–27 for surviving +/*T* ^*int2*^, 4–19 for non-surviving +/*T* ^*int2*^ and 5–49 for +/+). Weights of both sexes have been combined as no significant differences in the weights of males and females were found when considered as a percentage of the weight of wild-type siblings. Error bars show standard error of the means.

The +/*T* ^*ex1*^ mice were found at expected Mendelian frequencies at birth (49% of 370 neonates), failed to suckle, became inert, and died on the day of birth (Fig. 7B). They were noted to have arched backs, be of small size and were 80% of the weight of their wild-type siblings (*p* = 3.2 x 10^–12^; +/*T* ^*ex1*^ 1.193g ± 0.015 se, n = 19 and +/+ 1.511g ± 0.022 se, n = 16). Weight differences of embryos were found from 17.5 dpc when +/*T* ^*ex1*^ were 92.5% of the weight of their wild-type siblings (*p* = 0.021; +/*T* ^*ex1*^ 0.766 ± 0.019 se, n = 6 and +/+ 0.800g ± 0.015, n = 12). Thus +/*T* ^*ex1*^ mice exhibit considerable similarity in phenotype with MatDp(dist2) mice which have two maternally derived copies and no paternally derived copies of the *Gnas* cluster [23]. MatDp(dist2) mice are known to lack *Gnasxl* expression and have two expressed doses of *Gnas* in imprinted tissues.

The +/*T* ^*int2*^ mice were found at expected Mendelian frequencies at birth (45% of 303 neonates) but there was a severe shortage by weaning; only 45 (23%) of 199 weaners were +/*T* ^*int2*^ (*p* < 0.001, χ^2^ = 59.7, 1 df, 2-tailed). Although over 98.5% of deaths of +/*T* ^*int2*^ (65/66) occurred in the first two postnatal weeks, only 12% (8/66) occurred within two days of birth with over 50% (34/66) occurring between postnatal days 5 and 9. Those +/*T* ^*int2*^ that survived past weaning showed normal viability thereafter. Mice with paternal inheritance of +/*T* ^*int2*^ were observed to be smaller and leaner than their wild type siblings within a few days of birth (Fig. 7A). The +/*T* ^*int2*^ mice and wild-type siblings were weighed from birth until shortly after weaning (Fig. 7C). The +/*T* ^*int2*^ were smaller by 1 day (*p* = 0.001) and growth retardation became more pronounced over the next two weeks followed by some indication of recovery. The postnatal losses of +/*T* ^*int2*^ occurred during the period of growth retardation in the first two weeks and comparisons of surviving and non-surviving +/*T* ^*int2*^ between postnatal days 2 and 12 showed that the survivors were larger (*p* < 0.007) (Fig. 7C).

Previous studies have shown that neonatal mice that lack all *Gnasxl* transcripts have reduced suckling [6,23,29]. Given that the level of *Gnasxl* transcripts was severely reduced in +/*T* ^*int2*^ mice, suckling was investigated in +/*T* ^*int2*^ and wild-type littermates for up to a week after birth by daily visual assessment of the presence and size of a milk spot. Altogether 145 observations were made on 49 +/*T* ^*int2*^ and 182 observations on 47 +/+. Prominent milkspots were seen on at least one day in all 47 +/+ but only in 28 of 49 +/*T* ^*int2*^ (*p* < 0.0001, Fisher’s exact test, 2 tailed). Conversely a milk spot was absent or very small on one occasion or more in all 49 +/*T* ^*int2*^ but in only 6 of 47 +/+ (*p* < 0.0001, Fisher’s exact test, 2-tailed). Thus suckling appears to be compromised following paternal transmission of *T* ^*int2*^.

## DISCUSSION

**Fig 8:**
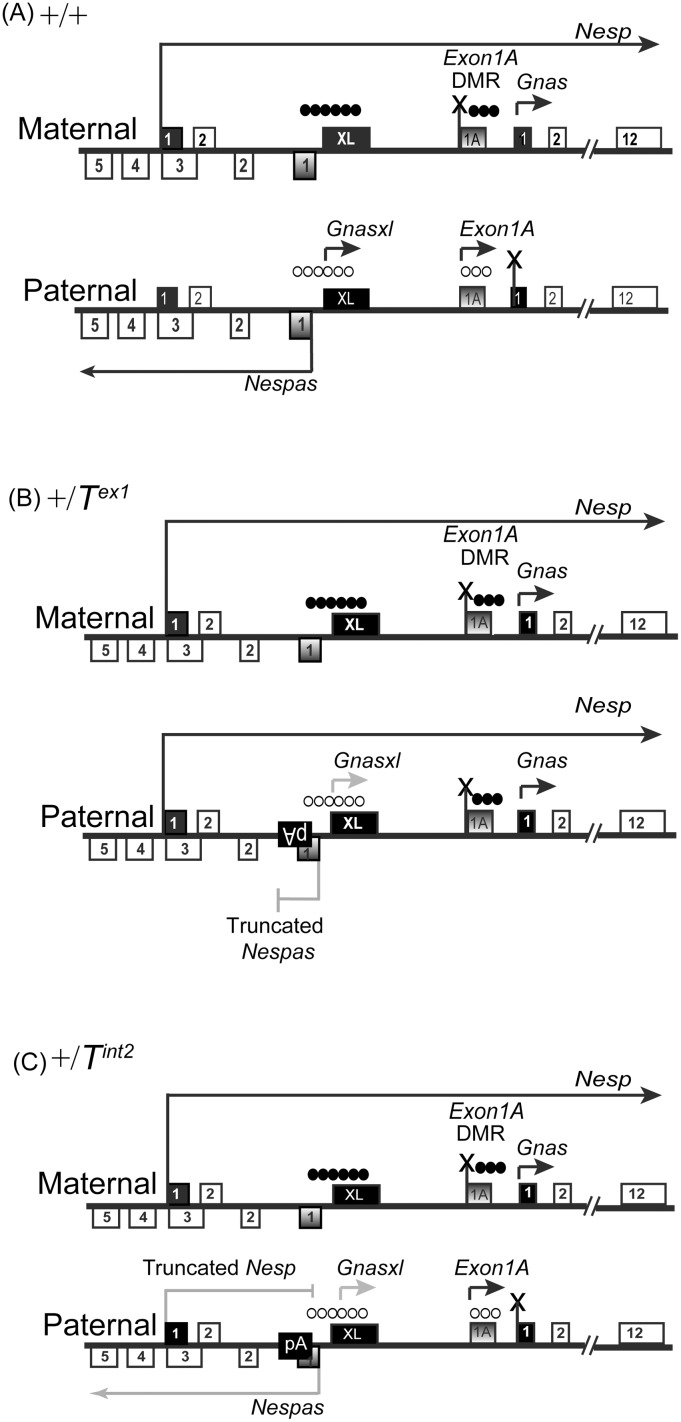
Composite of DMR methylation and transcript expression in (A) +/+, (B) +/*T* ^ex1^ and (C) +/*T* ^int2^. The solid black fill boxes represent first exons of the protein-coding transcripts *Nesp*, *Gnas* and *Gnasxl* whereas shaded boxes are first exons of the non-coding transcripts *Nespas* and *Exon1A*. A string of filled circles represents a methylated DMR, and a string of open circled represents an unmethylated DMR. ‘X’ shows that the corresponding transcript is repressed. Transcripts expressed at low levels are shown in grey.

We set out to investigate if ectopic expression of *Nesp* on the paternal allele is correlated with *de novo* methylation of the *Exon1A* DMR, and upregulation of *Gnas*. On paternal inheritance of the *T* ^*ex1*^ allele, *Nesp* was de-repressed, transcribed through the downstream *Exon1A* DMR which was methylated in neonatal brain (Fig. 8B). This ectopic methylation at the *Exon1A* DMR was absent in sperm of mutant males, present at 10.5 dpc in progeny of mutant males, and therefore must be acquired post-fertilisation. On paternal inheritance of the *T* ^*int2*^ allele, *Nesp* was weakly expressed and was truncated upstream of the *Exon1A* DMR. In this mutant, the *Exon1A* DMR was not ectopically methylated on the paternal allele (Fig. 8C). Previously, a gain of ectopic methylation was also seen in a deletion mutation +/*∆NAS-DMR*, in which a 1.6 kb region of the *Nespas-Gnasxl* DMR is deleted, *Nesp* is de-repressed on the paternal allele and is transcribed through the *Exon1A* DMR [24].

On maternal inheritance of the *T* ^*ex1*^ or the *∆NAS-DMR* mutations, expression of *Nesp* was unaltered, and the *Exon1A* DMR remained methylated [17,24]; whereas the *Exon1A* DMR lost methylation on maternal inheritance of the *T* ^*int2*^ allele on which *Nesp* is truncated upstream of the *Exon1A* DMR. Chotalia *et al*. (2009) [16] observed a similar loss of methylation at the maternally inherited *Exon1A* DMR in their *Nesp* truncation mutant (referred to as *Nesp* ^*trun*^), in which the poly-A cassette is inserted further upstream of the *Exon1A* DMR compared to the *T* ^*int2*^. Thus, a correlation of *Nesp* expression through the *Exon1A* DMR and methylation of the *Exon1A* DMR emerges on both parental alleles.

Intriguingly, while the *Exon1A* DMR is a germline DMR on the maternal allele, it acquires somatic methylation on the *T* ^*ex1*^ paternal allele: an acquisition that appears to be dependent upon *Nesp* transcription traversing through the intragenic *Exon1A* DMR.

Methylated CGIs are routinely found intragenic to actively transcribed genes in both plants and animals [18,30]. A correlation of CGI methylation and its location within active transcription units is also seen in both oocytes and male primordial germ cells, being significantly more prevalent in the oocyte [21,22,31,32]. However, that transcription is required for *de novo* methylation of intragenic CGIs has only been conclusively shown (for germline DMRs in imprinted clusters) in the female gamete so far [16]. It is not known whether a similarly causal relationship exists between transcription traversing CGI and acquisition of methylation at the CGI post-fertilization, in somatic cells. To our knowledge, ectopic methylation seen at the *Exon1A* DMR in 10.5 dpc mutant embryos, but absent in mutant sperm is the first example of transcription driven intragenic *de novo* CGI methylation in somatic cells, since it must be acquired post-fertilization.

The mechanisms of *Nesp* expression dependent methylation of the *Exon1A* DMR may be the same in both the oocyte and on paternal inheritance of the *T* ^*ex1*^ allele. As was proposed by Chotalia et al (2009) for the oocyte, *Nesp* expression may simply ‘open’ the chromatin at the *Exon1A* DMR on a paternally inherited *T* ^*ex1*^ allele thus making the latter accessible to *de novo* DNA methyltransferases [16]. Alternatively, methylation of the *Exon1A* DMR may result due to deposition of DNA methylation permissive histone modifications like H3K4me3 and H3K36me3 brought about by ectopic *Nesp* expression on the *T* ^*ex1*^ allele [16,21,33–39].

We hypothesize that the paternal ectopic methylation at the *Exon1A* DMR is acquired at the time of the genome wide wave of *de novo* methylation which first starts in the inner cell mass of the blastocyst and as the embryo implants, continues into early post-implantation development [40,41]. Indeed, most somatic DMRs studied to date first show *de novo* methylation post-implantation [42–45]. Robust *Nesp* expression is also first detected 6.5 dpc onwards in embryonic development (Mehta et al., in prep).

Our previous investigations showed that the *Nespas-Gnasxl* DMR is not methylated on the paternal allele of the *+/T* ^*ex1*^ mutants despite expression of *Nesp* which traverses the *Nespas*-*Gnasxl* DMR [17]. A similar absence of ectopic methylation is seen at the *Gnasxl* promoter region in a deletion mutation +/*∆NAS-DMR*, in which *Nesp* is fully expressed on the paternal chromosome and *Exon1A* DMR is ectopically methylated [24]. Thus, the susceptibility of the paternally inherited *Nespas-Gnasxl* DMR to *de novo* methylation due to *Nesp* expression appears to be different to that of the DMR in the oocyte: two maternal *Nesp* truncations described previously result in loss of methylation at both the *Exon1A* and the *Nespas*-*Gnasxl* DMRs, albeit the extent of lack of methylation at the *Nespas*-*Gnasxl* DMR is variable [16,17]. This implies that while *Nesp* expression traversing through the *Exon1A* DMR consistently induces methylation at the *Exon1A* DMR on maternal as well as on the paternal allele, methylation at the *Nespas*-*Gnasxl* DMR is only affected by expression of *Nesp* in the maternal gamete. One point of distinction between the two DMRs is that the *Nespas*-*Gnasxl* DMR encompasses the imprinting control region (ICR) of the *Gnas* cluster, controlling imprinting of all transcripts of the cluster; while despite being a germline DMR, the *Exon1A* DMR is not an ICR, and only controls imprinted expression of *Gnas*. An analysis of high-throughput data of histone modifications in ES cells showed that a combination of H3K4me3, H3K9me3 and H4K20me3 marks, found at all known germline DMRs that are ICRs, is indeed seen at the *Nespas*-*Gnasxl* DMR, but not at the *Exon1A* DMR [46]. Thus the two DMRs have distinct histone modifications which may result in differing susceptibility to *de novo* DNA methylation upon being transcribed through [31].

Although the *Nespas-Gnasxl* DMR was not methylated on the paternal allele of either the +/*T* ^*ex1*^ or the +/*T* ^*int2*^ mice, *Gnasxl* expression was drastically reduced in both mutants. Disruption of a *Gnasxl* enhancer element caused by insertion of the poly-A sequence may cause this repression. Alternatively, promoter competition between the *Nesp* and *Gnasxl* promoters for common transcription factors or enhancers could also result in reduction of *Gnasxl* expression when *Nesp* is de-repressed. Such promoter competition must be restricted to the paternal allele; mutants that inherit *Nesp* truncation alleles (*Nesp* ^*trun*^ and *T* ^*int2*^) maternally have de-repressed *Gnasxl* despite *Nesp* being expressed *in cis* [16,17].

Paternal inheritance of mutations that result in loss of *Gnasxl* expression gives rise to neonates with severely reduced suckling ability that become thin and inert on the day of birth, with the majority dying within a day or so of birth probably as a result of hypoglycaemia. A small proportion, up to 20% depending on genetic background, survive but are severely growth retarded and become small lean adults [6,29,47]. It is expected that deficiency of all proteins that use the XL exon occurs in *Gnasxl* nulls and is the cause of the phenotype. Taken together with work on other mutants it appears likely that lack of XLαs and/or XXLαs accounts for the small lean phenotype but not the suckling defect (Table 1).

**Table 1:** Mouse phenotypes resulting from gain of *Gnas* and/or loss of *Gnasxl*.

Mutation		MatDp(dist2) [Maternal duplication of distal chromosome 2]ΔNAS-DMR [Deletion of ICR]*T* ^*ex1*^ [Insertion of poly A cassette into Nespas exon 1]	*Gnasxl* knockout	Deletion of *Gnas* exon 2	*T* ^*int2*^ [Insertion of poly A cassette into *Nesp* intron 2]	*Sml* [ENU induced point mutation in *Gnas* exon 6]	*Ex1A-T* [Insertion of poly A cassette after *Exon1A]*
Inheritance		NA^a^	P^b^	P	P	P	P
Affected proteins		NESP↑, XLαs↓, XXLαs↓, XLN1↓, ALEX↓, Imprinted Gsα↑	XLαs↓, XXLαs↓, XLN1↓, ALEX↓	XLαs↓, XXLαs↓, XLN1↓, non-imprinted Gsα↓	XLαs↓, XXLαs↓, XLN1↓, ALEX↓	XLαs↓, XXLαs↓, non-imprinted Gsα↓	XLαs↓, XXLαs↓, XLN1↓, imprinted Gsα↑
Phenotype	Neonatal	Fail to suck, Narrow bodies, Inactive, 100% lethality in 24 hours	Severely reduced suckling, narrow bodies, inactive, most die in first few days after birth	Severely reduced Suckling, narrow bodies, inactive, most die in first few days after birth	Reduced suckling	Suckling unaffected	Suckling unaffected
	Perinatal to weaning	NA	Postnatal growth retardation, lean bodies, up to 20% survive to weaning	Postnatal growth retardation, lean bodies, up to 25% survival to weaning	Postnatal growth retardation, lean bodies, 23% survival to weaning	Postnatal growth retardation, lean bodies, 46% survival to weaning	Postnatal growth retardation, 85% survival to weaning
References		[17,23,24,50], this paper	[29]	[6,47]	[17], this paper	[12,49,51]	[48]

^a^ NA, not applicable

^b^ P, paternal

The neonatal phenotype in +/*T* ^*ex1*^ strongly resembles that observed in *Gnasxl* nulls indicating that much of the phenotype in +/*T* ^*ex1*^ can be attributed to severely diminished *Gnasxl* expression, probably disrupting expression of all XL proteins. However +/*T* ^*ex1*^ mice show even greater lethality than *Gnasxl* nulls, invariably dying within a few hours of birth. In addition to a very low level of *Gnasxl*, +/*T* ^*ex1*^ have other disparities in gene expression at the *Gnas* cluster with overexpression of both *Nesp* and *Gnas* in imprinted tissues ([17], this paper). Although the effects of a double dose of *Nesp* are not known, overexpression of *Gnas* is associated with post natal growth retardation [12,48,49]. The imbalanced expression of all three protein coding genes within the *Gnas* cluster may account for the complete neonatal lethality that occurs not only in +/*T* ^*ex1*^ but also in MatDp(dist2) on all genetic backgrounds tested (Peters *et al*., unpublished). In +/*T* ^*int2*^, expression of *Gnas* is unaltered, *Nesp* expression is slightly raised but, as in *T* ^*ex1*^, the level of *Gnasxl* is severely diminished and is likely to affect all XL proteins (Table 1). The very low levels of *Gnasxl* probably accounts for the phenotype observed. Following the neonatal period the +/*T* ^*int2*^ mice follow a growth trajectory typical of other deletion or loss of function *Gnasxl* mutants; a severe growth retardation over the first two weeks followed by some recovery [29,48,50].

There are now a number of mutants at the *Gnas* cluster including +/*T* ^*ex1*^ and +/*T* ^*int2*^ that indicate that appropriate expression of imprinted protein coding transcripts within the cluster is required for normal development and survival. Imprinted expression is primarily regulated by the parent specific expression of two RNAs, a noncoding antisense RNA, *Nespas*, on the paternal chromosome and its sense counterpart, a protein coding RNA, *Nesp* on the maternal chromosome.
